# Two cases of non-mucinous cystadenomas of the pancreas with pancreatobiliary phenotype and ovarian-like stroma

**DOI:** 10.1186/s40792-019-0673-y

**Published:** 2019-07-23

**Authors:** Shoko Yamashita, Tetsuya Ikemoto, Yuji Morine, Satoru Imura, Shuichi Iwahashi, Yu Saito, Shinichiro Yamada, Toshiaki Yoshimoto, Koichi Tsuneyama, Mitsuo Shimada

**Affiliations:** 1 Department of Digestive and Transplant Surgery, Tokushima University, 3-18-15 Kuramoto-cho, Tokushima, 770-8503 Japan; 2 Department of Pathology and Laboratory Medicine, Tokushima University, Tokushima, Japan

**Keywords:** Mucinous cystic neoplasm, Pancreatic neoplasms, Serous cystic neoplasm

## Abstract

**Background:**

Diagnosis of cystic tumor of the pancreas is based on the World Health Organization criteria that classify pancreatic cystadenomas into four types: intra-ductal papillary mucinous neoplasms, mucinous cystic neoplasms (MCNs), serous cystic neoplasms, and solid pseudo-papillary neoplasms depending on their secretion and presence of ovarian-like stroma. Recently, Albores-Saavedra identified non-mucinous cystadenomas of the pancreas with pancreato-biliary phenotype and ovarian-like stroma. This precipitated examination of the proportions of these rare tumors in patients treated at Tokushima University Hospital.

**Case presentation:**

Case 1 was a 40-year-old woman with a cystic tumor in the tail of the pancreas. Computed tomography (CT) revealed a diffuse and non-enhanced cystic tumor in the tail of the pancreas. This tumor was diagnosed as a simple cyst at this point. However, 2 years later, the tumor had increased in size by 3 cm. Thus, laparoscopic distal pancreatectomy was performed. The content of the cyst was serous. The epithelial cells were lined with a single layer of cuboidal cells and the tumor had ovarian-like stroma pathologically. The final pathological diagnosis was non-mucinous cystadenoma of the pancreas with ovarian-like stroma.

In Case 2, a cystic tumor in the pancreas was found by medical examination in a woman in her sixties who presented without symptoms. CT showed a 1.5-cm cystic tumor in the tail and body of the pancreas and a septum in the cyst. Nine years later, the tumor had grown to 2.4 cm in diameter and had a clear septum in the cyst. This tumor was diagnosed preoperatively as MCN. Thus, laparoscopic distal pancreatectomy was performed. The cyst contained serous fluid. Microscopic examination showed no ovarian-like stroma and the epithelial cells were lined by a single layer of cuboidal cells. The final pathological diagnosis was non-mucinous cystadenoma of the pancreas with ovarian-like stroma.

**Conclusions:**

Accurate preoperative diagnosis of this type of pancreatic cystic tumor may be difficult, although it occurs more often than expected. Non-mucinous cystadenomas of the pancreas with ovarian-like stroma need to be considered as a differential diagnosis.

## Background

The concept of cystic tumors in the pancreas is complicated. Several guidelines have been developed to classify these tumors and establish therapeutic criteria, such as observation and surgical resection [1–3]. However, even if these guidelines are followed, some cases can be difficult to classify into a particular type. Pancreatic cystic neoplasms (PCNs) are classified into four types: intra-ductal papillary mucinous neoplasms (IPMNs), mucinous cystic neoplasms (MCNs), serous cystic neoplasms (SCNs), and solid pseudo-papillary neoplasms depending on their secretions and the presence of ovarian-like stroma [4]. In some retrospective studies, malignant tumors derived from MCNs have accounted for 10–40% of all MCNs, and their malignant potential is higher than for the other three cystic tumors [5, 6], even though their prognosis is good [7]. Previously, resection was recommended for MCNs in all patients at the time of diagnosis. However, more recently, only MCNs with a high risk of becoming malignant have been resected [1–3].

Regarding MCNs, Albores-Saavedra proposed a new classification of non-mucinous cystadenomas of the pancreas with pancreato-biliary phenotype and ovarian-like stroma and researched nine cases of this type of tumor [8]. This tumor does not secrete mucin but has ovarian-like stroma and characteristics that are different from other MCNs, SCNs, and IPMNs. As described above, the malignant potential is higher in MCNs than in other types of tumors, and thus it is important to differentiate precisely between the types of cystic tumors from the point of view of recurrence. Therefore, this study retrospectively reviewed cystic tumors resected in Tokushima University Hospital, Japan, between 2006 and 2018, and the frequency and features of these rare tumors that have non-mucinous secretion with ovarian-like stroma.

## Patients and methods

### Case selection

**Fig. 1:**
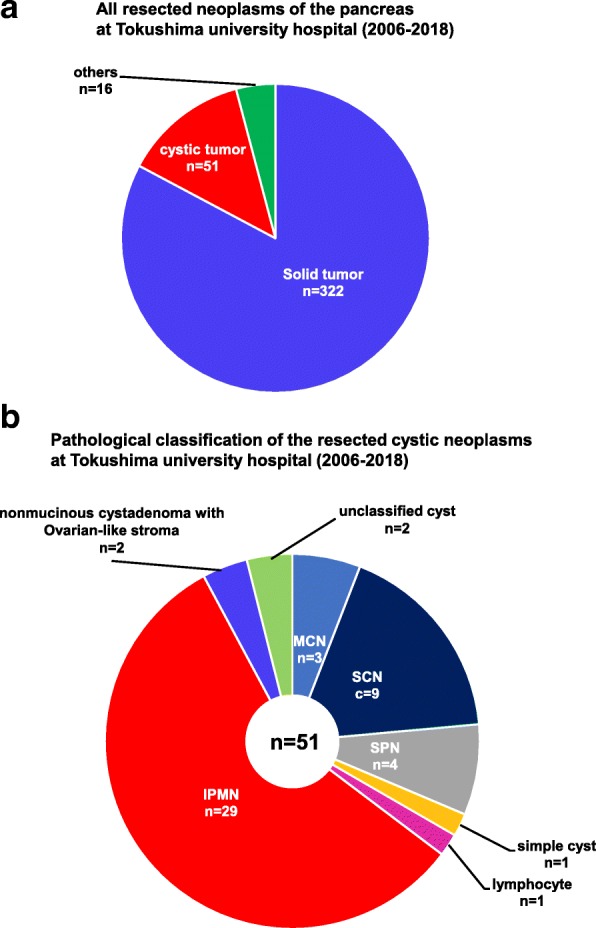
Ratio of respective tumor patterns in resected tumors of the pancreas at Tokushima University Hospital (2006–2018)

All resection cases were identified by searching the pathology database of Tokushima University Hospital. After exclusion of IPMNs (29 cases) and malignant tumors (322 cases), we identified 22 patients with a cystic tumor of the pancreas who underwent surgical resection at Tokushima University Hospital between 2006 and 2018. The tumors were diagnosed pathologically as MCN (3 cases), SCN (9 cases), SPN (4 cases), simple cyst (1 case), lymphocyst (1 case), and unclassified cyst (2 cases). The other two cases (9%) were non-mucinous and had ovarian-like stroma (Fig. 1a, b). Both cases were middle-aged women and their cysts had enlarged gradually over several years; however, the cysts were diagnosed as benign because of a lack of malignant features. This report describes these two cases of non-mucinous cystadenomas of the pancreas with pancreato-biliary phenotype and ovarian-like stroma.

This study was approved by the Ethics Committee of Tokushima University (TOCMS ID 3215). Written informed consent was obtained from all the patients.

### Histological examination

Initial investigations focused on whether the tumors were MCNs by detecting mucin-producing cells using hematoxylin and eosin (HE) staining. Patients with MCNs were then excluded from those who had undergone resection of cystic tumors of the pancreas. Among the other non-mucinous cystic tumors, those that had no ovarian-like stroma were diagnosed as SCNs and those that had ovarian-like stroma were diagnosed as non-mucinous cystadenomas with ovarian-like stroma. As the next step, the ovarian-like stroma and mucin-producing cells were investigated by immune staining.

### Immunohistochemistry

All samples were fixed in 10% buffered formalin and embedded in paraffin. De-paraffinized sections were heat treated and incubated with primary antibodies as a first step. The slides were then stained for mucin core protein (MUC)1, MUC2, MUC5AC, MUC6, cytokeratin (CK)7, CK20, estrogen receptor (ER), progesterone receptor (PgR), and inhibin with antibodies as follows: anti-MUC1 (Ma695;Novocastra, Newcastle, UK), MUC2 (Ccp58; Novocastra ), MUC5AC (CLH2; Novocastra), MUC6 (CLH5; Novocastra ), CK7 (M7018; Dako, Glostrup, Denmark), CK20 (M7019; Dako), ER (SP1; Ventana Medical Systems, Tucson, AZ, USA), PgR (1E2; Ventana Medical Systems), and inhibin (M3609; Dako). At least two pathologists diagnosed these tumors.

## Case presentation

### Case 1

**Fig. 2:**
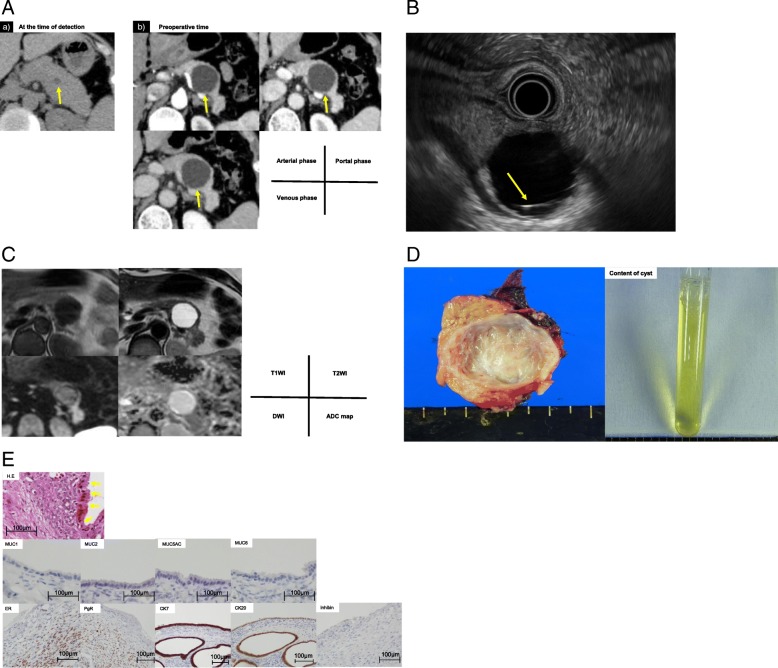
**a** Abdominal CT at the time of detection (a) and preoperative CT (b) revealed low-density cystic tumor in the tail of the pancreas in all phases (arrow), but no septum or nodules were detected in the cyst. **b** Endoscopic ultrasound showed a cystic tumor with a septum (arrow). **c** Magnetic resonance imaging showed a low-intensity T1 signal and high-intensity T2 signal in the tumor. **d** The resected cyst specimen contained serous and yellow fluid. **e** Microscopic examination showed that the tumor was a unilocular cyst and had a thin septum. The epithelial cells were lined with a single layer of cuboidal pancreato-biliary cells (arrow) and had no sign of malignancy. The cellular ovarian stroma was present beneath the epithelium with hematoxylin and eosin staining. Immuno-pathologically, the tumor showed nuclear expression of an estrogen receptor and a progesterone receptor in the stromal cells, and the epithelial cells showed no expression of MUC1, 2, 5AC, and 6, which showed that the tumor had an ovarian-like-stroma but not MUC secretion. CT computed tomography, MUC mucin

A 40-year-old woman was referred to our hospital because of a cystic tumor in the tail of the pancreas found by medical examination. She had no symptoms. Laboratory tests were all within normal limits: carcinoembryonic antigen (CEA) 0.4 ng/mL, carbohydrate antigen (CA)19-9 11 U/mL, duke pancreatic monoclonal antigen type 2 (DUPAN-II) < 25 U/mL, and s-pancreas antigen-1 (Span-1) 6 U/mL. Computed tomography (CT) revealed a 0.7-cm diffuse and non-enhanced cystic tumor in the tail of the pancreas but no septum or nodules in the cyst (Fig. 2a). Endoscopic ultrasonography (EUS) showed suspicious structure of the septum but no nodules in the cyst (Fig. 2b). Magnetic resonance imaging (MRI) showed that the tumor had a low-intensity signal on T1-weighted imaging (WI) and high-intensity signal on and T2-WI, which revealed water-like content (Fig. 2c). All images showed that the connection between the cyst and main pancreatic duct was indistinct. The tumor was diagnosed as a simple cyst at that time. However, 2 years later, the tumor had increased by 2.6 cm to become 3.3 cm in diameter. The tumor was then diagnosed as SCN and considered at that time to be a simple cyst with MCN as the differential diagnosis. Thus, laparoscopic distal pancreatectomy was performed. The operation time was 283 min and the intraoperative blood loss was 137 mL. Severe adhesion near the splenic hilum was considered to have been caused by pancreatitis. Thus, it was decided that the spleen could not be preserved safely, and splenectomy was also performed. The content of the cyst was serous and did not include mucin or yellow fluid (Fig. 2d). The resected specimen showed that the tumor was a unilocular cyst and had a thin septum (Fig. 2d). The epithelial cells were lined with a single layer of cuboidal pancreato-biliary cells and did not show signs of malignancy. HE staining showed that the cellular ovarian-like stroma was present beneath the epithelium. No malignant or invasive lesions were found. Immuno-pathologically, the tumor showed nuclear expression of ER and PgR but not inhibin in the stroma cells (Fig. 2e). The epithelial cells showed no expression of MUC1, 2, 5AC, and 6, which showed that the tumor had ovarian-like stroma but not mucin secretion (Fig. 2e). The final pathological diagnosis was non-mucinous cystadenoma of the pancreas with pancreato-biliary phenotype and ovarian-like stroma. The patient’s postoperative course was uneventful, and she remains well 12 months postoperatively with no evidence of tumor recurrence.

### Case 2

**Fig. 3:**
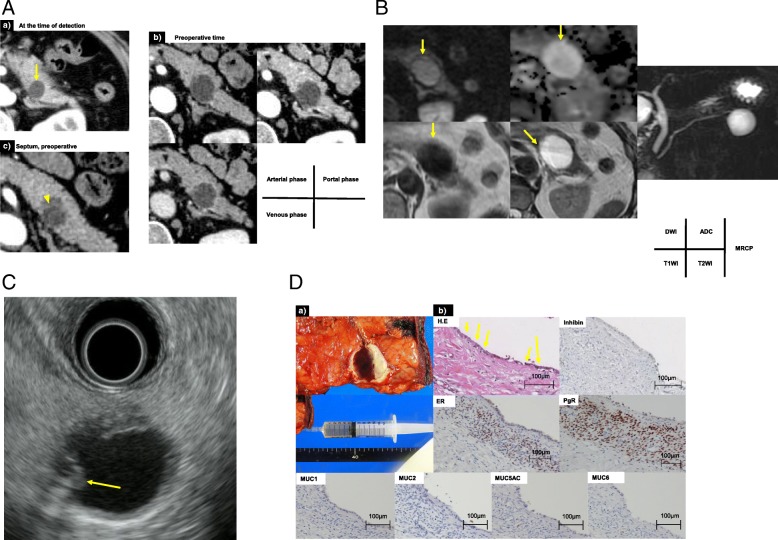
**a** CT showed a 1.5-cm cyst with a septum at the time of detection (a). Preoperative CT showed a 2.4-cm low-density cystic tumor in the tail of the pancreas in all phases and showed a septum (b, c). **b** Magnetic resonance imaging showed a low-intensity T1 signal and high-intensity T2 signal in the tumor (arrow), and the connection between the pancreatic duct and cystic tumor could not be detected by magnetic resonance cholangiopancreatography. **c** Endoscopic ultrasound showed a thickened cell wall in the cyst (arrow). **d** (a) Resected specimen and cyst content. (b) Microscopy showed no ovarian-like stroma and epithelial cells lined by a single layer of cuboidal pancreato-biliary cells (arrow). However, the stromal cells expressed an estrogen receptor and a progesterone receptor. The epithelial cells showed no expression of MUC1, 2, 5AC, and 6, which showed that the tumor had an ovarian-like stroma but not MUC secretion. No malignant and invasive lesion was found. CT computed tomography, MUC mucin

A cystic tumor was found by medical examination in the pancreas of a woman in her sixties who presented without any symptoms. Laboratory tests were all within normal limits: CEA 1.3 ng/mL, CA19-9 9 U/mL, and DUPAN-II < 25 U/mL. CT showed a 1.5-cm cystic tumor in the tail and body of the pancreas and a septum in the cyst, and the tumor was suspected to be MCN. Observation was selected as the treatment of choice after obtaining informed consent. However, 9 years later, the tumor had grown to 2.4 cm in diameter and there was a clear septum in the cyst. This tumor was not enhanced in all phases (Fig. 3a). MRI showed low intensity on T1-WI and high intensity on T2-WI, and magnetic resonance cholangiopancreatography (MRCP) did not reveal a connection between the main pancreatic duct and the cystic tumor (Fig. 3b). EUS showed a thickened cell wall in the cyst (Fig. 3c). This tumor was diagnosed preoperatively as MCN. Thus, laparoscopic distal pancreatectomy with splenectomy was performed. The operation time was 260 min and intraoperative blood loss was 175 mL. The cyst contained serous fluid (Fig. 3d). Microscopic examination showed no ovarian-like stroma and the epithelial cells were lined by a single layer of cuboidal pancreato-biliary cells (Fig. 3d, arrow). Although the stromal cells expressed ER and PgR, no malignant or invasive lesions were found. The epithelial cells showed no expression of MUC1, 2, 5AC, and 6 (Fig. 3d). The final pathological diagnosis was non-mucinous cystadenoma of the pancreas with pancreato-biliary phenotype and ovarian-like stroma. The patient remains well 15 months postoperatively with no evidence of tumor recurrence.

## Discussion

Most PCNs are detected incidentally when abdominal imaging is performed for other indications [9]. PCNs are categorized pathologically using the World Health Organization (WHO) classification [4]. There are four types, which have varying malignant potential: IPMNs, MCNs, SCNs, and solid pseudo-papillary neoplasms. MCNs have ovarian-like stroma that is strongly immunostained for ER, PgR, and inhibin, but the other cystic tumors are not stained. Thus, the presence of ovarian-like stroma is essential for diagnosis of MCNs. Findings associated with malignant transformation in MCNs include large size (5 cm or larger in one series), a thickened or irregular cyst wall, an internal solid component or mass, and possible calcification of the cyst wall [5, 6, 10]. Previously, all MCNs were recommended to be resected based on their malignant potential. However, recently, a recommendation for resection is considered appropriate only for MCNs with malignant features such as positive cytology for a malignant neoplasm; a mucinous cyst ≥ 3 cm; Kras and/or guanine nucleotide-binding protein (G protein); α stimulating activity polypeptide (GNAS) mutation with tumor protein 53 (TP53) and phosphatidylinositol-4,5-bisphosphate 3-kinase catalytic subunit α (PIK3CA); or phosphatase and tensin homolog deleted from chromosome (PTEN) mutation by molecular testing [2, 4]. In this study, surgery was performed in both cases because the tumor size was around 3 cm and showed enlargement [1–3].

There are three major guidelines regarding the treatment of MCNs, provided by The International Association of Pancreatology [1], The American Gastroenterological Association [2], and European experts’ consensus statements [3]. These guidelines propose different treatments for MCNs as well as follow-up plans after surgery, and these differences cause confusion regarding the management of patients with MCNs. Depending on the global accumulation of MCN cases, a more concrete management algorithm is required.

Albores-Saavedra found that nine cases could have been non-mucinous cystadenomas of the pancreato-biliary phenotype and ovarian-like stroma during a review of 31 cases of tumors that were diagnosed as mucinous cystadenomas [8]. All the patients were female with a mean age of 45 years. Seven tumors were symptomatic and tumor markers were within normal limits in all cases. Microscopically, the epithelial cells were lined by a single layer of cuboidal cells, and 2–3% of the epithelial cells included goblet cells. The septa of the cysts included spindle cells that resembled ovarian-like stroma. No dysplasia was found in either of the cases in the present study or in any of the cases reported by Albores-Saavedra. Although non-mucinous cystadenomas of the pancreato-biliary phenotype and ovarian-like stroma are insufficient evidence to determine the prognosis, it is suggested that it is better than for MCNs and SCNs, considering their pathological features. Albores-Saavedra reported nine cases (29%) with ovarian-like stroma but no mucin-producing cells, while the current study found two cases (9%). In fact, some tumors like these might be diagnosed as other typical cystic tumors, such as MCNs, SCNs, and simple cysts. For example, in Case 2, the tumor was initially diagnosed as SCN pathologically, because the cyst was filled with serous fluid, the stroma was not stained by inhibin, and the presence of spindle cells was not clear. WHO classification of the sensitivity of each immunostaining test regarding ovarian-like stroma is reported as follows: ER 30% and PgR 60–90% [4]. The Japanese Multi-institutional Retrospective Observation Study of MCNs reported the sensitivity as follows: ER 78% and PgR 88% [11]. There is no report of the sensitivity of the inhibin immunostaining rate. Albores-Saavedra reported that all the spindle cells expressed ER and PgR but only five of eight cases (62.5%) expressed inhibin [8]. These results suggest that PgR is the most useful tool to investigate ovarian-like stroma. When it is difficult to use HE staining to evaluate ovarian-like stroma, staining the cystic tumor using PgR is required or the use of multiple tools such as PgR plus ER.

When it is difficult to diagnose the type of cystic tumor preoperatively, EUS–fine-needle aspiration (FNA) is often reported to be a useful tool [7, 11, 12]. In the present study, CT and MRI showed no septa or nodules in the cysts but EUS could detect the septa. However, EUS has low sensitivity and EUS–FNA has a high risk of tumor dissemination, and sometimes it is difficult to diagnose pathologically because of the low number of cells. Recently, EUS-guided through-the-needle forceps biopsy (EUS–TTNFB) has been performed as a new diagnostic tool for cystic tumors [11]. In the DETECT study, which investigated the utility of EUS–TTNFB, the sensitivity of dual through-the-needle imaging (cytology and needle-based, confocal laser-induced endomicroscopy) of pancreatic cysts was about 100%. Some complications such as pancreatitis have been observed; however, it is expected to become a safer procedure in the next few years. It is anticipated that a useful tool like this will be available in the future for accurate preoperative diagnosis of cystic tumors.

## Conclusions

We have reported two rare cases of non-mucinous cystadenomas of the pancreas with ovarian-like stroma in the stromal tissue. Accurate preoperative diagnosis may be difficult; however, this type of pancreatic cystic tumor occurs more often than expected and its prognosis is better than that of other cystic tumors of the pancreas.

## Data Availability

The datasets supporting the conclusions of this article are included within the article and its additional files.

## Abbreviations

CA19-9: Carbohydrate antigen 19-9
CEA: Carcinoembryonic antigen
CT: Computed tomography
ER: Estrogen receptor
EUS: Endoscopic ultrasonography
EUS-TTNFB: EUS-guided through-the-needle forceps biopsy
FNA: Fine-needle aspiration
G protein: Guanine nucleotide-binding protein
GNAS: α stimulating activity polypeptide
HE: Hematoxylin and eosin
IPMN: Intra-ductal papillary mucinous neoplasm
MCN: Mucinous cystic neoplasms
MRCP: Magnetic resonance cholangiopancreatography
MRI: Magnetic resonance imaging
MUC: Mucin core protein
PCN: Pancreatic cystic neoplasms
PgR: Progesterone receptor
PIK3CA: Phosphatidylinositol-4,5-bisphosphate 3-kinase catalytic subunit alpha
PTEN: Phosphatase and tensin homolog deleted from chromosome
SCN: Serous cystic neoplasm
Span-1: S-pancreas-1 antigen
TP53: Mutation with tumor protein 53
WHO: World Health Organization
WI: Weighted image
